# Sleep, Insomnia and Hypnotics

**Published:** 1892-05

**Authors:** 


Sleep, Insomnia and Hypnotics. By E. P. Hurd, M. D., Member of the Massachusetts Medical Society; Member of the Climatological Society; Member of the Société de Medicine Pratique (Paris, France); one of the Physicians in the Anna Jaques Hospital, Newburyport, Mass. The Physician's Leisure Library. George S. Davis, Detroit, Mich. 1891. Price, cloth, 50 cents; paper, 25 cents.

To successfully treat insomnia is frequently one of the most perplexing duties of the physician. He must master all the idiosyncrasies of his patient, not only in so far as they pertain to his morbid condition, but also with reference to the effect upon his economy that the various drugs have. He must control the methods and habits of life of the insomnolent victim, and he must in many ways assume a knowledge of human nature in its multifarious aspects and deviations from the normal line. In this book will be found many valuable hints that will lead to a solution of the difficult problem of inducing sleep when it will not come unaided, and we think that the author has handled the matter with wisdom and discretion. It is a readable little volume, and deals with the subject in a masterly manner. A not unfrequent classical quotation adds much to the pleasure of its reading.

